# Understanding the role of oncogenic human papillomavirus (HPV) status on adherence behaviors among women with abnormal cervical cytology

**DOI:** 10.1186/s12905-020-01168-2

**Published:** 2021-01-18

**Authors:** Catriona Buick, K. Joan Murphy, Doris Howell, Kelly Metcalfe

**Affiliations:** Faculty of Health, York University, Health, Nursing and Environmental Studies Building, Room: 301A, 4700 Keele St, Toronto, ON M3J 1P3 Canada

**Keywords:** Human papillomavirus, Adherence behaviors, Cervical screening abnormalities, Psychosocial health, Cervical cancer screening, Colposcopy

## Abstract

**Background:**

With the introduction of oncogenic Human Papillomavirus (HPV) testing into cervical screening there is a renewed focus on primary prevention among high-risk groups. To date, little is known about the effectiveness of this program, and the extent to which individual-level factors, such as psychosocial health and agency, may play a role. In particular, it is unclear if knowledge of one’s oncogenic HPV status impacts on adherence behaviors amongst women with screening abnormalities. The purpose of this study was to identify if clinical, demographic or psychosocial factors predict non-adherence with recommended colposcopy follow-up.

**Methods:**

This prospective pilot study included 145 women referred to a large Toronto colposcopy clinic between December, 2013 and September, 2014. Demographic, clinical and psychosocial characteristics were collected at three points in time: (1) at initial colposcopy consultation; (2) 4–6 weeks following initial consultation, and; (3) at time of follow-up appointment (within 12 months of initial consultation).

**Results:**

Overall, 13% (n = 145) of the women were classified as non-adherent. Older women (OR = 0.73, *p* < 0.01) and those with higher-grade lesions (OR = 0.10, *p* < 0.01) were less likely to be non-adherent, whereas current smokers (OR = 22.46, *p* < 0.01) were more likely to be non-adherent. While not statistically significant, variation in rates of non-adherence amongst the various HPV status groups (untested; 15.3%, HPV positive; 5.3%, HPV negative; 6.7%) warrants further study.

**Conclusion:**

Findings of this study indicate that younger women, those with higher-grade lesions and current smokers were more likely to be non-adherent to recommended colposcopy follow-up. While HPV status did not reach statistical significance, the direction of this finding suggests that testing for HPV may have a positive reinforcing role on adherence to follow-up. The direction of this finding warrants further study, and potentially a practical clinical goal as HPV testing for women becomes standard of care.

## Background

A number of modifiable risk factors have been implicated in the progression of oncogenic HPV infection to cervical cancer [1]. Failure to adhere with follow-up recommendations for management of premalignant cervical abnormalities [2] is among the most notable. Adherence to recommended cervical screening guidelines is associated with better clinical outcomes, with an inverse association between exposure to screening and stage of cancer at diagnosis. While adhering to recommendations for the management of abnormal cytology is proven to decrease the incidence of cervical cancer, adherence rates remain sub-optimal in women with premalignant abnormalities [3]. Non-adherence has significant implications for the effectiveness of cervical screening programs and cancer prevention [4] as well as for the financial burden of potentially preventable disease.

A number of socio-demographic, psychosocial and clinical factors, as well as health system issues are known to influence adherence to recommended follow-up in women with screening abnormalities [5–8], including: reminder calls or text messages prior to the patients appointment [9, 10]; the use of video colposcopy during the physical examination [11]; mail-out educational materials [12]; transportation incentives [6]; and; educational outreach [12]. To date, interventions targeting such individual barriers and risk factors have not eliminated non-adherence in this population, and suggest that other non-measured factors (or yet to be discovered factors) may provide a broader understanding of adherence behaviors.

In some jurisdictions, HPV testing has been used among women with low-level cytologic changes as a triage test to distinguish those who are at risk of cervical cancer (HPV + ve) from those who are not (HPV –ve) [13]. Receiving a positive oncogenic HPV test result is known to increase women’s psychosocial burden [14–16]. While among the public, knowledge of the virus is generally poor [17], those who undergo testing and have knowledge of their HPV status have a greater understanding of HPV and its causes and consequences [18]. In turn, women attending for *routine* cervical cytology screening with greater HPV health literacy are more likely to adhere to follow-up [19] and to experience less psychosocial burden [16],. Yet, with the introduction of oncogenic HPV testing into current screening and colposcopy practices, it is unclear if knowledge of one’s HPV status affects adherence to recommended follow-up behaviors amongst those with screening abnormalities. If it can be established that knowledge of one’s oncogenic HPV status has a positive impact on adherence to follow-up in this population, this evidence can further support the implementation of HPV testing. The purpose of this research was two-fold: (i) to investigate the association between knowledge of one’s oncogenic HPV status (positive, negative or untested) and adherence to recommended follow-up, and; (ii) to identify clinical, demographic, and psychosocial factors associated with adherence to recommended follow-up after colposcopic assessment following cervical screening abnormalities.

## Methods

### Participant recruitment and follow-up

This exploratory study included adult women with an abnormal cervical screening cytology who attended the colposcopy clinic at two large academic institutions in Toronto between December 2013 and September 2014. Eligible participants were between the ages of 18 and 70 years, had sufficient English proficiency to provide informed consent and complete study measures. Women were ineligible if they had a previous HPV test, were pregnant, had a previous history of a genital HPV-related cancer or were previous patients at this clinic. All participants were followed until the data collection was completed, except in the following circumstances: it was determined that a malignancy was present; the participant transferred their care; or the patient was discharged after the initial consultation.

All participants provided written informed consent. Data was subsequently collected from each participant via survey and chart audit at three points in time: (1) at initial consultation at the colposcopy clinic (Time 0); (2) at 4–6 weeks following the initial consultation where participants received test results, including HPV if applicable (Time 1), and; (3) at time of follow-up appointment (within 12 months of initial consultation) (Time 2) (See Additional file 1: Fig. 1). Participants received standard of care for their abnormal cervical screening abnormality and were not asked to attend the clinic outside of their routine care.

After the initial consultation, participants were asked to phone the clinic at a predetermined date/time (4–6 weeks after) to receive their test results (standard of care at organization) over the telephone. If a participant failed to connect, a follow-up protocol was in place for the nurse to connect with the participant and if after two weeks the participant was unable to be informed of their test results they were considered a true loss to follow-up.

Using an estimated non-adherence of 25%, a sample of 380 participants with complete follow-up data would be required for 80% power and a two-sided alpha of 0.05.

### Definition of non-adherence

The primary outcome for the current analysis was “Non-adherence”, which was defined as not attending the first scheduled clinic appointment for follow-up or treatment after the colposcopy consultation. The recommended follow-up appointment was determined after the initial colposcopy consultation and was a minimum of 6 weeks to a maximum of 12 months after initial consultation for either the treatment or follow-up. The time point Time (2) varied for each participant from 1.5 months to 12 months post initial consultation depending on physician recommended follow-up.

### Psychosocial, clinical, and demographic predictors

Predicted and exploratory variables were comprised of psychological, socio-demographic and clinical characteristics and determined using a combination of chart-abstracted data and self-reported questionnaires (Additional file 1: Fig. 2).

### At time (0) initial colposcopy consultation

*State anxiety* was measured using the Spielberger State Anxiety Inventory Six item short form [20]. The six-item short-form has reliability coefficient of (α = 0.82) and was combined to result in an overall prorated score ranging from 20 to 80 [20]. A clinical cut-off (≥ 40) was also applied to the baseline anxiety scores (State Anxiety Inventory) for this sample to indicate clinically detectable or significant anxiety [21].

### At time (1) receipt of results 4–6 weeks after initial consultation

*HPV Burden* was measured using the self-administered HPV Impact Profile (HIP) tool [22]. The scale includes a 29-item questionnaire divided into 7 domains: (worries and concerns, emotional impact, sexual impact, self-image, partner issues and transmission, interaction with doctors and control and life impact). The response for each item is a point-scale (0–10) visual analog scale [22] and raw scores from all 7-domains are totaled from the questionnaire and are transformed to a 0–100 scale. The HIP has a Cronbach’s alpha ranging from 0.64 to 0.90 for the seven domains. It has demonstrated construct validity by ascertaining known group differences and convergence [22].

*Knowledge of oncogenic HPV* score was determined using a 9-item scale with true–false responses adapted from Kahn & colleagues [23–25]. The original 13-item scale was developed to identify variables associated with intention to receive the HPV16/18 vaccine and therefore aimed specifically at the oncogenic types of HPV infections [23]. The intraclass correlation coefficient measuring test—retest reliability of the original scale was 0.95 and Cronbach was 0.78 [25]. The correct responses were used to calculate an overall knowledge score for each participant.

*Perceived risk of cervical cancer* was measured by Likert scale responses to questions examining the participant’s perceived personal risk (*“How likely do you think it is that you will develop cervical cancer in the future?”;* 1 = ”very low” to 5 = ”very high”), perceived comparative risk (*“Compared to the average person your age, would you say that you are: less likely or more likely to develop cervical cancer in the future?”;* 1 = ”less likely” to 3 = ”more likely”) and two questions on perceived severity of cervical cancer (*“Cervical cancer is a serious disease that can be deadly, and; Cervical cancer is impossible to recover from”;* 1 = ”strongly disagree” to 5 = ”strongly agree”) based on the Risk-Perception Attitude Framework [26–28]. These items were combined into an overall measure of risk perception [28] with higher total scores indicating a higher perceived risk of cervical cancer.

The *Impact of Event Scale* [29] was used to measure current subjective distress in relation to being at risk of cervical cancer. The scale consists of 15 items (seven intrusion items and eight avoidance items) [30]. Participants were asked to rate the frequency of the intrusive and avoidant emotions using a four-point scale (0 = “not at all”; 1 = “rarely”; 2 = “sometimes”; 3 = “often”) which allows for total score calculations (intrusive 0–35 and avoidance 0–40), with larger scores indicating greater stress responses [30]. The measure has shown a high degree of internal consistency using Cronbach’s Alpha (intrusion = 0.78, avoidance = 0.82) [29]. A clinical cut-off (≥ 33) was utilized for cancer-related distress, as a score of 33 or more has been used as a threshold for diagnosis of Post-Traumatic Stress Disorder (PTSD) [31].

Self-efficacy was tailored to a behavior-specific unit of analysis [32]. It was operationalized by asking about confidence in the ability of the recommended behavior in preventing cancer, assessing perceived difficulty in following through with the recommended behavior, and confidence in the ability to perform the recommended behavior [33]. The self-efficacy tool included a confidence scale ranging from 0 to 100 with preliminary instructions to establish the appropriate mindset and a specified time set to determine perceived capability of maintaining a given level of functioning at the follow-up appointment [32]. Individual scores were added to produce an overall average self-efficacy score for each participant.

### Statistical analysis

Participant socio-demographic and clinical characteristics were analyzed using descriptive statistics for all study variables. Independent t-tests and chi-square tests (Fishers Exact) were used to examine demographic differences between participants and women who declined participation, and between those who were lost to follow-up. Next, a bivariate screening (α = 0.20) procedure was undertaken in order to mitigate the small sample size and ensure that relevant variables were initially explored and limited to those considered scientifically and clinically relevant. Finally, with only significant associations at the bivariate level included the final multivariable model, a conservative significance level (α = 0.05) was applied. Logistic regression (binary) analysis was subsequently used to estimate the relationship between individual predictors and the odds of non-adherence. All analyses were conducted in SPSS ® (version 20.0). Ethics approval was granted from the University Health Network Ethics Board (TASHN #13-5900-CE), Women’s College Hospital (#2014-0003-E) and the University of Toronto (#29239).

## Results

A total of 186 women were included in this study. For participants who only completed baseline questionnaires, 17.7% (n = 186) were classified as non-adherent (results not shown). Overall, a total of 145 women completed questionnaires for all three time points (69%). For the 145 participants who completed all study measures, 13.1% (n = 19) were classified as non-adherent (Table 1).

**Table 1:** Demographic and clinical characteristics of participants

Characteristics	n=145
*Adherence*	
No	19 (13.1%)
Yes	126 (86.9%)
Socio-demographics	
Age in years, mean (SD)	34.9±13.0
*Age*	
<30	67 (46.2%)
≥30	78 (53.8%)
*Income CDN ($)*	
≤34,999	30 (20.7%)
≥35,000	104 (71.7%)
Decline	11 (7.6%)
*Education*	
High school or less	14 (9.7%)
University/college	131 (90.3)
*Born in Canada*	
No	39 (26.9%)
Yes	106 (73.1%)
Years in Canada*	23.1±18.4
*Relationship status*	
Married/steady	89 (61.4%)
Single	56 (38.6%)
*Smoking status*	
No	121 (83.4%)
Yes	24 (16.6%)
Medical characteristics	16 (11.0%)
*Lesion severity (post-results)*	
Low-risk abnormalities	83 (57.2%)
High-risk abnormalities	62 (42.8%)
*Previous history of colposcopy *	
No	106 (73.1%)
Yes	38 (26.2)
Unknown	1 (0.7%)
*Sexually transmitted infections*	
None	115 (79.3%)
Chlamydia/herpes	24 (16.5%)
Other/unknown	6 (4.2%)
*Contraception use*	
No	50 (34.5%)
Yes	94 (64.8%)
Condoms/OCP	78 (83.0%)
Other	16 (17.0%)
Video colposcopy**	
No	112 (89.0%)
Yes	25 (17.2%)
Unknown	8 (5.5%)
*Immunosuppression*	
No	129 (89.0%)
Yes	16 (11.0%)
*HPV vaccine*	
No	115 (79.3%)
Yes	28 (19.3%)
Unknown	1 (0.7%)
Gardasil	26 (96.3%)

N (%) for categorical variables; μ ± SD for continuous variables *N = 37 N = 137 **(video colposcopy); otherwise N = 145; Low risk abnormalities include: normal, LSIL, ASCUS; High risk abnormalities include: AGC, HSIL/CIS, AIS, ASC-H

Characteristics for the 145 participants are shown in Tables 1 and 2. This sample was comprised of younger women (mean age was 34.9 years), with lower grade abnormalities (57.2%), and a high rate of contraception use (64.8%). The sample reported high levels of income (71.4% with ≥ $35,000), education (90.3% with university of college education), and social support (61% being married or in a steady relationship). Overall, 26.9% of participants were born outside Canada, with most being longer-term immigrants (mean time in country: 23 years). Finally, the sample reported a high baseline state anxiety score with 58.6% of participants indicating a clinically detectable level of significant anxiety.

**Table 2:** Psychosocial and knowledge characteristics

Characteristic	n = 145
Baseline	
State anxiety score	44.9±13.9
*State anxiety clinical cut-off*	
<40 ≥40	60 (41.4%) 85 (58.6%)
*1st degree relative history of cancer*	
No Yes Unknown	109 (75.2%) 34 (23.4%) 2 (1.4%)
*1st degree relative died of cancer*	
Yes	9 (6.2%)
Post-results	
HPV Burden*	37.5 ±17.6
Self-efficacy	84.5 ±15.0
Cancer-related distress	22.4 ±16.8
*Cancer-related distress clinical cut-off*	
< 33 ≥ 33	104 (71.7%) 41 (28.3%)
Perceived risk of cancer	11.6 ±2.2
Knowledge of HPV (total)	7.4 ±1.2

N = 128 for HPV Burden; otherwise N = 145 (%) for categorical variables; μ ± SD for continuous variables

After disclosure of results, HPV Knowledge [mean score: 7.4 ± 1.2 (range 4–9)] and self-efficacy scores [84.5 ± 15 (range 27–100)] among the study population was high. Forty-one women (28.3%) met or exceeded the clinical cut-off for the cancer-related distress for a diagnosis of PTSD (≥ 33) (Table 2). Although the pattern did not reach statistical significance, the prevalence of non-adherence tended to be highest amongst those who were untested for HPV (15.3%), compared to HPV positive (5.3%) and HPV negative (6.7%) groups (Table 3.).

**Table 3:** Frequency of HPV status by non-adherence to follow-up

	Adherence No	Adherence Yes	χ^2^	p value
*HPV status*				
Negative Positive Untested	1 (6.7%) 1 (5.3%) 17 (15.3%)	14 (93.3%) 18 (94.7%) 94 (84.7%)	1.42*	0.52

### Predictors of non-adherence

Initial bivariate screening revealed significant differences in age, immigration status, smoking status, lesion severity, type of follow-up, and state anxiety with non-adherence behaviors (Table 4). The final multivariable model therefore included oncogenic HPV status—the key hypothesized clinical predictor for in adherence behaviors—alongside the aforementioned predictors. The resulting multivariable logistic regression revealed that younger women (OR = 0.73, 95% CI 0.61–0.88, *p* < 0.01), those with lower grade abnormalities (OR = 0.10, 95% CI 0.00–0.30; *p* < 0.01) and current smokers (OR = 22.46, 95% CI 2.16–233.01, *p* < 0.01) were more likely to be non-adherent (Table 5). Interactions between age and lesion severity did not reach significance and were therefore excluded from the final model.

**Table 4:** Bivariate relationship between clinical psychosocial and demographic characteristics and non-adherence to recommended follow-up

	Odds ratio	95% CI	*p* value
Clinical characteristics			
*HPV status*			
Positive Negative Untested	1.00 (ref) 0.39 0.31	– 0.05–3.20 0.04–2.46	– 0.38 0.27
*Lesion severity (post results)*			
Low-grade High-grade	1.00 (ref) 0.13	– 0.03–0.58	– **0.01**
*History of colposcopy*			
No Yes	1.00 (ref) 1.77	– 0.64–4.89	– 0.27
*Video colposcopy*			
No Yes	1.00 (ref) 0.49	– 0.10–2.25	– 0.36
*Type of follow-up*			
Follow-up Treatment	1.00 (ref) 0.23	– 0.06–0.84	– **0.03**
Knowledge of HPV	1.20	0.77–1.86	0.41
*Reminder calls*			
No Yes	1.00 (ref) 1.50	– 0.53–4.42	– 0.43
Psychosocial characteristics			
*State anxiety scale (baseline)* *(clinical Cut-off)*			
< 40 ≥ 40	1.00(ref) 2.17	– 0.74–6.39	– **0.16**
Self-efficacy	1.00	0.97–1.04	0.82
Perceived risk of cancer	1.00	0.80–1.25	0.98
*Cancer related distress (clinical Cut-off)*			
< 33 ≥ 33	1.00 0.89	– 0.30–2.66	– 0.84
HPV Burden	0.99	0.96–1.10	0.46
Demographic characteristics			
Age (years)	0.78	0.67–0.90	** < 0.01**
*Born in Canada*			
No Yes	1.00 (ref) 7.77	– 1.00–60.33	– **0.05**
*Education*			
High school or less University/college	1.00 (ref) 2.07	– 0.25–16.80	– 0.50
*Income CDN ($)*			
≤ 34,999 ≥ 35,000	1.00 (ref) 1.29	– 0.34–4.85	– 0.71
*Relationship status*			
Married/steady Single	1.00 (ref) 1.51	- 0.57–4.00	- 0.40
*Smoking status*			
No	1.00 (ref)	–	
Yes	2.77	0.93–8.22	− **0.07**
*1st degree relative with cancer*			
No	1.00 (ref)	–	
Yes	0.56	0.15–2.06	− 0.39

**Table 5:** Multivariable* adjusted odds of non-adherence to follow-up

	Odds ratio	95% CI	p value
Clinical characteristics			
*HPV status*			
Positive			
Negative Untested	1.00 (ref) 1.24 1.62	– 0.11–14.31 0.12–21.13	– 0.87 0.71
*Lesion severity (post results)*			
Low-grade High-grade	1.00 (ref) 0.10	– 0.00–0.30	– ** < 0.01**
*Type of follow-up*			
Follow-up Treatment	1.00 (ref) 3.91	– 0.30–50.73	– 0.30
Psychosocial characteristics			
State anxiety scale (baseline) (clinical cut-off)			
< 40 ≥ 40	1.00 (ref) 1.21	– 0.30–4.87	– 0.79
Demographic characteristics			
Age (years)	0.73	0.61–0.88	** < 0.01**
*Born in Canada*			
No Yes	1.00 (ref) 5.35	– 0.52–55.3	– 0.16
*Smoking status*			
No Yes	1.00 (ref) 22.46	– 2.16–233.01	– ** < 0.01**

## Discussion

Findings of this study suggest that non-adherence to recommended follow-up exists for women diagnosed with abnormal cytology. Specifically, younger women, those with lower-grade lesions, and those who were current smokers were more likely to be non-adherent to the recommended follow-up after referral for abnormal cytology. This work is novel in its approach to examine the consequences of knowledge of one’s HPV status by HPV-DNA testing in the cervical screening population. Importantly, non-adherence was highest amongst those who were untested for HPV, compared to those who were tested for HPV (positive or HPV negative). Although this finding did not reach statistical significance, the direction of this finding provides a basis for further study as a practical clinical goal, as HPV testing for women becomes standard of care.

In this study, rates of non-adherence for women attending colposcopy with cervical screening abnormalities ranged from 13.1 to 17.7% considerable lower than the existing literature (20–50%) [3, 5, 6, 34–36]. Differences between these and our study were adherence was defined as attendance at recommended follow-up after initial colposcopy consultation and our study was conducted in a universal health care system. Cross comparison of non-adherence rates can be problematic due to differing definitions and methods for examining adherence across samples as well as geographic location and sociodemographic differences. This sample was comprised of younger women (< 40), with lower grade abnormalities, a high rate of contraception use, and high baseline state anxiety, which is similar to the broader colposcopy population [36]. A relatively large proportion of our sample reported higher levels of income, education, and social support—with 61% of the sample either reporting being married or in a steady relationship. These factors have often been associated with attendance at screening or follow-up [6, 37], and could account in part for the lower rate of non-adherence found in this study. Furthermore, the sample includes only English speakers, meaning that further exploration of rates (and predictors of) non-adherence amongst new immigrants with language barriers who may have been excluded from the current pilot study is necessary.

In our initial examination of adherence behaviors in this study, a number of key predictors were identified. First, it was observed that women who had a lower-grade lesion severity and were younger, were less likely to be adherent. In unadjusted models, women returning for treatment were more likely to adhere than women returning for routine follow-up. This relationship was no longer significant once adjusted for confounders, suggesting that it may be mediated in part by lesion severity, as higher-grade lesion is associated with adherence, and women with high-grade lesions would be asked to return for treatment. Second, smoking status also reached statistical significance in that women who were current smokers were less likely to adhere. While this finding is consistent with the literature [38], given the small number of cases, these findings must be interpreted with caution. Nonetheless, a higher rate of smoking might reflect a generally lower endorsement of healthy behaviors and a lack of awareness of an increased risk for cervical cancer [39]. It also highlights the need for a comprehensive baseline assessment of smoking history in this population, which could be used to help tailor interventions towards women at higher-risk.

With respect to screening-related behavior, we found higher rates of non-adherence amongst those who were untested for HPV (untested: 15.3%; HPV negative: 6.7% and; HPV positive: 5.3%), which suggests that testing for HPV-DNA may have a positive reinforcing role on treatment adherence. This pilot work is particularly critical in light of emerging Expert Panel recommendations for the use of primary HPV testing in the cervical screening setting in Ontario [13], indicating that all screening aged women will receive HPV testing in the near future. Further research is needed to examine the HPV status and adherence relationship and the potential to target at-risk groups of women to encourage adherence with colposcopy recommendations.

In contrast to the main hypothesis and previous work [7, 40], perceived risk of cervical cancer scores and HPV knowledge were not related to adherence in this study. This can be explained in part by a number of study-specific factors, such as the mode of recruitment, changes in provincial cervical screening guidelines, and sample size constraints. Importantly, women in our study received a comprehensive assessment and educational session regarding HPV at their initial colposcopy consultation, which was completed prior to their HPV knowledge questionnaire. This type of education has been shown to increase HPV knowledge for at least two weeks [41]. Higher levels of education are also associated with greater HPV awareness [42], a possible confounder in our study; given that 90.3% of participants who completed our baseline knowledge questionnaire had a University or College education. Further differences include the discrepancy between a woman’s intention to return for cervical screening and their ultimate follow through. This has led to inconsistencies in the relation between women’s knowledge of their HPV and changes in their perceived risk of developing cervical cancer wherein young women who perceive themselves to be highly susceptible to a cervical cancer diagnosis indicated greater intent on returning for cervical screening [26], yet, when followed longitudinally, *intention* to return for follow-up was not consistently associated with actual follow-up adherence behaviors [19, 26]. Furthermore, among those who actually returned, they were less likely to perceive their likelihood of dying to be high [19]. Higher adherence to screening recommendation has also been seen among women with a family history or a genetic mutation for breast cancer [43, 44].

Finally, relatively little is currently known about how HPV burden impacts on adherence. Consistent with previous literature, we demonstrated a moderate association between HPV burden and anxiety (r = 0.44); however, neither anxiety nor HPV burden were related to adherence. Previous work has found that anxiety is a predictor of non-adherence in women who receive inadequate cytology results [45] or are asked to return for follow-up [7, 8]. Women who do not attend for colposcopy follow-up have been shown to have greater state anxiety than women who do, and differences in anxiety amongst women with various HPV statuses is well established [7, 8, 14, 15, 18, 46–48]. Because women who are HPV positive have higher levels of anxiety, one might expect to see higher rates of non-adherence in the HPV positive group, a finding that may be moderated by lesion severity [46]. Further work is therefore necessary to understand the inter-relation between HPV knowledge, anxiety, and adherence behaviors in a prospective setting.

## Limitations

A number of potential limitations warrant mention. First, differences in adherence behavior and educational attainment were found between those who completed the psychosocial questionnaires at time (1) (n = 145) versus those who did not (n = 41) (post-hoc analysis, Additional file 1: Table 1). Specifically, those who were not adherent to recommended follow-up were significantly less likely to complete the psychosocial questionnaire (T1) [14 (42.4%)] compared to n = 19 (57.6%) of the final analytic sample (n = 145, resulting in a healthier than expected selected sample which potentially underestimates the rate of non-adherence to follow-up). Second, the majority of unanswered questions tended to be those that were more sensitive in nature, such as questions related to sexual intercourse and feeling satisfied with their current sex life. Third, the use of bivariate screening in model building has been criticized, as it tailors to the specific study sample, and is consequently less generalizable [49]. Moreover, a number of structural changes may have contributed to lower-than-expected patient volumes, including: changes in screening guidelines; increase in HPV vaccine use; relocation of the Colposcopy center to a new hospital, and; changes to HPV triage approach over the study period). As a result, study recruitment was closed after 18 months, well short of the required sample size of 380. A post-hoc power analysis with the analytic sample (n = 145) suggests that the main analysis was underpowered (1 − β = 0.15), but nonetheless provides preliminary insight on recruitment feasibility on which to plan future multicenter studies. Finally, because of the prospective nature of the study, participants were fully aware that their adherence behaviors were being observed, which may have contributed to higher than expected rates of adherence [50].

## Conclusion

Taken together, results from this study reinforce existing evidence of factors related to non-adherence (e.g. age, lesion severity, and smoking) and generates new hypotheses for future study (e.g. HPV testing and HPV burden and self-efficacy). As the use of primary HPV testing is set to change the cervical screening landscape, this study provides pilot data and descriptive evidence to guide further research and clinical guidelines in regards to preventative behaviors and psychosocial factors in the colposcopy setting. Given the complex health needs of this population of women, these findings provide preliminary insight into the importance of knowing one’s HPV status, and groundwork for further psychosocial and behavior study into the outcomes associated with knowing the results of a HPV-DNA test for women.

## Supplementary Information

**Additional file 1.** **Supplemental Figure 1.** Prospective Study Design. **Supplemental Figure 2.** Overview of Study Variables and Measures. **Supplemental Table 1.** Missing Value Comparison.

## Data Availability

The data generated or analyzed during this study is included in this published article or available from the corresponding author on reasonable request.

## Abbreviations

HPV: Human Papillomavirus
HIP: Human Papillomavirus Impact Profile
PTSD: Post-Traumatic Stress Disorder
