# Supplementary material for: Understanding the role of oncogenic human papillomavirus (HPV) status on adherence behaviors among women with abnormal cervical cytology
Source: BMC Womens Health. 2021 Jan 18;21:29. doi: 10.1186/s12905-020-01168-2 (PMC7812645; doi:10.1186/s12905-020-01168-2)
Supplement: Supplementary file 1 — Additional file 1. Supplemental Figure 1. Prospective Study Design. Supplemental Figure 2. Overview of Study Variables and Measures. Supplemental Table 1. Missing Value Comparison. [file 12905_2020_1168_MOESM1_ESM.docx]

**Supplemental Figure 1.** Prospective Study Design


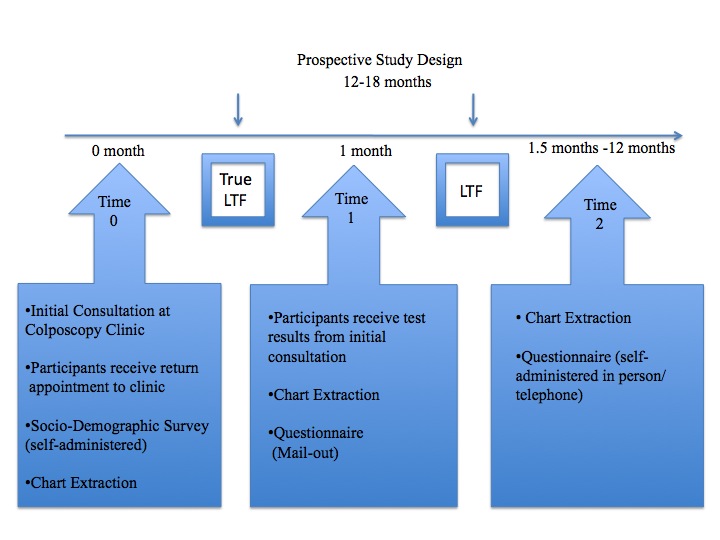


**Supplemental Figure 2.** Overview of Study Variables and Measures

| Variable | Measure(s) |
| --- | --- |

| **Time (0) Initial Consultation** | |
| --- | --- |
| Age | Chart Audit |
| Education | Socio-demographic Questionnaire |
| Income | Socio-demographic Questionnaire |
| Lesion Severity | Chart Audit |
| Length of Time in Canada | Socio-demographic Questionnaire |
| Family History of Cancer | Socio-demographic Questionnaire |
| Video Colposcopy Use | Clinician Report |
| Relationship Status | Socio-demographic Questionnaire |
| State Anxiety | State Anxiety Inventory Six Item Short Form^20^ |
| Smoking Status | Socio-demographic Questionnaire |
| HPV vaccine practices | Socio-demographic Questionnaire |
| Type of Follow-up | Chart Audit |

| **Time (1) 4-6 weeks following Initial Consultation** | |
| --- | --- |
| Psychosocial Burden | Questionnaire, Human Papillomavirus Impact Profile (HIP)^22^ |
| Cancer Risk Distress | Questionnaire, Impact of Event Scale^29^ |
| Knowledge of HPV | Questionnaire, 9-item knowledge score^25^ |
| Self-efficacy | Questionnaire, 3 self-reported questions Likert Scale (5-points). Combined for overall score |
| Risk Perception of Cervical Cancer | Questionnaire, 4 self-reported questions Likert scale Combined for overall score |

| **Time (2) Follow-up** | |
| --- | --- |
| Adherence | Chart Audit |
| Smoking Status | Socio-demographic Questionnaire |
| HPV vaccine practices | Socio-demographic Questionnaire |

**Supplemental Table 1.** Missing Value Comparison

|  | **Analytic Sample**  (N=145) | **Excluded Sample***  (N=41) | **p-value** |
| --- | --- | --- | --- |
| ***Clinical Characteristics*** | | |  |
| Adherence  No  Yes | 19 (13.1%)  126 (86.9%) | 14 (34.1%)  27 (65.8%) | **<0.01** |
| HPV Status  Negative  Positive  Untested | 15 (10.3%)  19 (13.1%)  111 (76.6%) | 6 (14.6%)  8 (19.5%)  27 (65.8%) | 0.38 |
| Lesion Severity  Low-grade  High-grade | 83 (57.2%)  62 (42.8%) | 24 (58.5%)  17 (41.5%) | 0.88 |
| History of Colposcopy  No  Yes | 106 (73.1%)  38 (26.2%) | 32 (80.0%)  8 (20.0%) | 0.41 |
| Video Colposcopy  No  Yes | 112 (81.8%)  25 (18.2%) | 32 (91.4%)  3 (8.6%) | 0.19 |
| Type of Follow-up  Follow-up  Treatment | 86 (59.3%)  59 (40.7%) | 31 (76.6%)  10 (24.4%) | 0.06^┼^ |
| ***Psychosocial Characteristics*** | | |  |
| State Anxiety Scale (Clinical Cut-off)  <40  ≥40 | 60 (41.4%)  85 (58.6%) | 16 (41.0%)  23 (59.0%) | 0.97 |
| ***Demographic Characteristics*** | | |  |
| Age (years) | 34.9±13.0 | 31.9±9.6 | 0.17 |
| Born in Canada  No  Yes | 39 (26.9%)  106 (73.1%) | 14 (35.0%)  26 (65.0%) | 0.32 |
| Education  High School or less  University/College | 14 (9.7%)  131 (90.3%) | 9 (22.5%)  31 (77.5%) | **0.03** |
| Income CDN ($)  ≤34,999  ≥35,000 | 30 (22.4%)  104 (77.6%) | 9 (25.7%)  26 (74.3%) | 0.68 |
| Relationship Status  Married/Steady  Single | 89 (61.4%)  56 (38.6%) | 24 (60.0%)  16 (40.0%) | 0.87 |
| Smoking Status  No  Yes | 121 (83.4%)  24 (16.6%) | 30 (75.0%)  10 (25.0%) | 0.22 |
| 1^st^ Degree Relative History of Cancer  No  Yes | 109 (76.2%)  34 (23.8%) | 32 (80.0%)  8 (20.0%) | 0.62 |

*N=41 with missing Psychosocial Factor information taken at T(1), who were subsequently excluded, Bold indicates p<0.05 between groups using Chi-Square (categorical) or independent t-tests (continuous). ^┼^indicates a trend (0.05>p<0.10)
